# Mechanisms Underlying the Anti-inflammatory and Immunosuppressive Activity of Ruxolitinib

**DOI:** 10.3389/fonc.2019.01186

**Published:** 2019-11-07

**Authors:** Elena Maria Elli, Claudia Baratè, Francesco Mendicino, Francesca Palandri, Giuseppe Alberto Palumbo

**Affiliations:** ^1^Hematology Division and Bone Marrow Transplant Unit, Ospedale San Gerardo, ASST Monza, Monza, Italy; ^2^Department of Clinical and Experimental Medicine, Section of Hematology, University of Pisa, Pisa, Italy; ^3^Hematology Unit, Department of Hemato-Oncology, Ospedale Annunziata, Cosenza, Italy; ^4^Institute of Hematology “L. and A. Seràgnoli”, Sant'Orsola-Malpighi University Hospital, Bologna, Italy; ^5^Dipartimento di Scienze Mediche, Chirurgiche e Tecnologie Avanzate “G.F. Ingrassia”, University of Catania, Catania, Italy

**Keywords:** ruxolitinib, JAK inhibitors, immune system, immunosuppression, myeloproliferative neoplasms (MPNs), natural killer (NK) cells, dendritic cells, T cells

## Abstract

The JAK-STAT signaling pathway plays a central role in signal transduction in hematopoietic cells, as well as in cells of the immune system. The occurrence in most patients affected by myeloproliferative neoplasms (MPNs) of driver mutations resulting in the constitutive activation of JAK2-dependent signaling identified the deregulated JAK-STAT signal transduction pathway as the major pathogenic mechanism of MPNs. It also prompted the development of targeted drugs for MPNs. Ruxolitinib is a potent and selective oral inhibitor of both JAK2 and JAK1 protein kinases. Its use in patients with myelofibrosis is associated with a substantial reduction in spleen volume, attenuation of symptoms and decreased mortality. With growing clinical experience, concerns about infectious complications, and increased risk of B-cell lymphoma, presumably caused by the effects of JAK1/2 inhibition on immune response and immunosurveillance, have been raised. Evidence shows that ruxolitinib exerts potent anti-inflammatory and immunosuppressive effects. Cellular targets of ruxolitinib include various components of both the innate and adaptive immune system, such as natural killer cells, dendritic cells, T helper, and regulatory T cells. On the other hand, immunomodulatory properties have proven beneficial in some instances, as highlighted by the successful use of ruxolitinib in corticosteroid-resistant graft vs. host disease. The objective of this article is to provide an overview of published evidence addressing the key question of the mechanisms underlying ruxolitinib-induced immunosuppression.

## Introduction

The majority of patients affected by classic Philadelphia chromosome-negative (or *BCR-ABL1*-negative) myeloproliferative neoplasms (MPNs), including polycythemia vera (PV), essential thrombocythemia (ET), and myelofibrosis (MF), harbor mutations of the genes encoding for Janus kinase 2 (JAK2), thrombopoietin receptor gene (MPL), or calreticulin (CALR) (1, 2). Among patients with primary myelofibrosis (PMF), ~50% carry a *JAK2* mutation, 35% a *CALR* mutation, and <10% an *MPL* mutation (3, 4). By contrast, among patients with PV, *JAK2* mutations are predominant (>95%) (4). These so-called “driver mutations” which are mutually exclusive, result in constitutively activated JAK2 signaling and upregulation of JAK-signal transducer and activator of transcription (STAT) target genes (2, 4). The JAK-STAT signaling pathway plays a central role in normal hematopoiesis by mediating signals from a variety of cytokines and hematopoietic growth factors, in hematopoietic stem cells (1). It is also crucial for cytokine activation and signaling in the immune system (5, 6). As a consequence, patients with MPNs, and particularly MF, exhibit both uncontrolled myeloproliferation and abnormally elevated levels of circulating proinflammatory cytokines causing disease-related systemic symptoms (7).

Four members of the JAK family of kinases are recognized (JAK1, JAK2, JAK3, and TYK2). The JAK family plays a pivotal role in myeloid and lymphoid cell proliferation and differentiation and are differentially activated by different cytokines and growth factors. For example, the JAK2 protein kinase is primarily associated with the hematopoietic growth factors erythropoietin and thrombopoietin, and mediates the process of differentiation, proliferation, and avoidance of apoptosis; the JAK1 isoform is involved in the signaling pathway of proinflammatory cytokines such as IL-2, IL-6, and TNF-alpha (1). Interdependence between JAK family members is common (5).

The discovery in MPNs patients of mutations affecting JAK2 signaling has led to the identification of deregulated signaling through the JAK-STAT pathway as a major pathogenic mechanism of MPNs and prompted the development of drugs targeting JAK2 (8–11). Ruxolitinib (SB1518) was the first JAK2 inhibitor to be granted approval and reached the market in the US and Europe in 2011. This drug is a potent and selective oral inhibitor of both JAK2 and JAK1 protein kinases (IC50, 2.8 nM, and 3.3 nM, respectively). It is currently indicated for the treatment of patients with intermediate- or high-risk MF, including PMF, post-polycythemia vera, and post-essential thrombocythemia MF (PPV/PET-MF). Ruxolitinib is also indicated for the treatment of patients with PV who have had an inadequate response or are intolerant to cytoreductive therapy with hydroxyurea (12, 13). The drug is not specific for the mutated form of JAK2 and inhibits both the wild-type and JAK2V617F.

The efficacy of this compound in a larger cohort of patients with MF was demonstrated at first on 2 phase III randomized studies (14, 15), Controlled Myelofibrosis Study with Oral JAK Inhibitor Treatment (COMFORT-1 and COMFORT-II). The anti-JAK2 inhibitory action is responsible for the efficacy of ruxolitinib on the control of myeloproliferation, reducing splenomegaly and, in some cases, the JAK2V617F allele burden (16–18). The inhibition of wild-type JAK2 protein results also in myelosuppression, primarily expressed as anemia and thrombocytopenia, and less frequently by neutropenia, which rarely leads to drug discontinuation. The anti-JAK1 inhibitory action is responsible for the reduction of pro-inflammatory cytokines, with a consequent improvement of symptoms, quality of life and, ultimately, bone marrow fibrosis (19, 20). At the same time, the anti-cytokine action could potentially cause an immunosuppressive effect of the drug, since the immune system and the hematopoietic system share intracellular signaling pathways, mediated by common receptors for cytokines and growth factors which, by acting on the JAK-STAT pathway, are important for the proliferation, differentiation, and activation of cells involved in innate and adaptive immune responses. Indeed, relatively high rates of infectious complications and of hematological and solid tumors have been observed in ruxolitinib-treated patients (21–23). Immunomodulatory properties, on the other hand, may be beneficial in some instances as highlighted by the use of ruxolitinib in graft vs. host disease (GVHD) (24–28).

The key question as to what the immunological targets of JAK1/2 inhibition are is far from being solved, and data elucidating the mechanisms of ruxolitinib-induced immunosuppression are still limited. The objective of this article is to provide an overview of published evidence addressing this question. We will first discuss the data that have suggested and confirmed an immunosuppressive action exerted by ruxolitinib; we will then briefly review the available studies that have attempted to dissect the mechanisms underlying ruxolitinib-induced immunomodulation.

## Immunosuppressive Activity of Ruxolitinib

In the registrative COMFORT I trial, grade 3 and 4 neutropenia were recognized in 7.1 and 2% of patients in the ruxolitinib and placebo arm of the study (15). More recently, 5-year follow-up of COMFORT II trial noted grade 3 or 4 neutropenia or leukopenia in 8.9 and 6.3% of ruxolitinib-treated patients (29). Unfortunately, leukocyte subpopulations and functions or antibody deficiency were not documented in both studies. Theocharides et al. (30) recognized that homozygous calreticulin mutations in PMF lead to acquired myeloperoxidase deficiency and consequential neutrophil reduced efficiency.

The possibility that ruxolitinib may increase the susceptibility to infections in patients with MF was first suggested by the results of the COMFORT-I and COMFORT-II trials. In the COMFORT-I study (15), patients treated with ruxolitinib had higher rates of bacterial and herpes zoster infections than patients treated with placebo. The incidence of urinary tract infections and herpes zoster was 10.5 and 2.1%, respectively in the first 12 weeks of therapy, 6.7 and 3.5% between 12 and 24 weeks, 7.7 and 3.4% between 24 and 36 weeks. At 5 years of follow-up (18), the most severe infections were pneumonia and sepsis, which occurred at similar rates in patients treated with ruxolitinib or placebo. Over time, herpes zoster infections occurred at a higher rate in patients treated with ruxolitinib compared with placebo, but in the majority of cases these were single episodes of grade 1 or 2. Septic events were reported as a cause of death in 6 ruxolitinib arm patients, 1 in the placebo arm and 4 placebo arm patients with subsequent cross-over.

In the COMFORT-II study (29), most infections were grade 1 or 2. Pneumonia was the only serious infectious adverse event reported (1% in the ruxolitinib group vs. 5% in the best available therapy group). In the final 5-year analysis, with a median ruxolitinib exposure time of 2.6 years, herpes zoster infections (11.5%), pneumonia (13.1%), sepsis (7.9%), and urinary tract infections (24.6%) were reported, with only two confirmed cases of tuberculosis (1%). In both COMFORT studies, the incidence of severe infections in patients treated with ruxolitinib was similar to that observed in patients in the control group. A summary of exposure-adjusted rates of immunosuppressive events during long-term treatment with ruxolitinib in the COMFORT studies is presented in Table 1.

**Table 1:** Common immunosuppressive events during long-term treatment with ruxolitinib: Data from the final 5-year analyses of the COMFORT I and COMFORT II trials.

	**Ruxolitinib, randomized (*****n*** **=** **155)**		Ruxolitinib, crossover^*^ **(*****n*** **=** **111)**		**Best available therapy (*****n*** **=** **151)**	
	**All grades**	**Grade 3 or 4**	**All grades**	**Grade 3 or 4**	**All grades**	**Grade 3 or 4**
**COMFORT I (Verstovsek S et al. J Hematol Oncol 2017; 10:55)**						
Upper respiratory tract infection	8.5	0	9.5	0	15.5	1.0
Urinary tract infection	7.5	1.0	6.7	1.2	6.9	1.0
Pneumonia	7.2	5.1	7.1	3.2	10.7	7.8
Herpes zoster	3.5	0	5.8	0.4	1.0	0
Bronchitis	3.1	0	4.5	1.2	1.9	0
Nasopharyngitis	3.1	0	3.5	0	9.1	0
Sinusitis	2.6	0.2	2.8	0	2.9	1.0
Cellulitis	2.1	0.4	1.1	0	1.9	0
Influenza	1.7	0	1.1	0.4	0	0
Sepsis	1.7	1.7	1.5	1.5	1.9	1.0
Tooth abscess	1.5	0.2	1.5	0	0	0
Oral herpes	1.3	0	0.7	0	1.9	0
Skin infection	1.1	0	1.1	0	1.0	0
Viral infection	1.1	0	0.8	0	0	0
Viral gastroenteritis	0.9	0	0.4	0	1.9	0
Diverticulitis	0.8	0.2	1.1	0.4	1.9	0
Ear infection	0.8	0	1.5	0	0	0
Fungal infection	0.8	0	0.7	0.4	1.9	0
Localized infection	0.8	0	0.4	0.4	1.0	0
Lower respiratory tract infection	0.8	0	0.4	0	1.9	1.0
Septic shock	0.4	0.4	1.1	1.1	0	0
	**Ruxolitinib, randomized (*****n*** **=** **146)**		Ruxolitinib, crossover^*^ **(*****n*** **=** **45)**		**Best available therapy (*****n*** **=** **73)**	
	**All grades**	**Grade 3 or 4**	**All grades**	**Grade 3 or 4**	**All grades**	**Grade 3 or 4**
**COMFORT II (Harrison CN et al. Leukemia 2016; 30:1701-7)**						
Bronchitis	10.0	NR	3.8	NR	9.0	NR
Nasopharyngitis	9.8	NR	5.0	NR	13.4	NR
Pyrexia	9.5	NR	10.0	NR	10.5	NR
Anemia	NR	7.6	NR	15.1	NR	7.5
Thrombocytopenia	NR	4.9	NR	11.3	NR	6.0
Pneumonia	NR	2.4	NR	1.3	NR	6.0
Herpes zoster	3.9	NR	6.3	NR	0	NR
Gastroenteritis	4.2	NR	1.3	NR	1.5	NR
Urinary tract infection	4.6	NR	8.8	NR	3.0	NR
Cystitis	3.7	NR	1.3	NR	4.5	NR

*Data are exposure-adjusted rates per 100 patient years, regardless of relationship to study drug*.

* *Patients randomized to best available therapy were allowed to crossover to receive ruxolitinib after 6 (COMFORT I) or 12 (COMFORT II) months in response to protocol-defined disease progression*.

*COMFORT, Controlled Myelofibrosis Study with Oral JAK Inhibitor Treatment; NR not available*.

In the JUMP study (an open-label phase 3b expanded-access trial which enrolled 2,233 patients from 26 countries, without access to ruxolitinib outside of a clinical study), a preliminary analysis of 1,144 intermediate and high-risk patients showed that the incidence of infections was low: pneumonia (5.3%), urinary tract infections (6%), and nasopharyngitis (6.3%). Tuberculosis was reported in five patients (0.3%); the reactivation of hepatitis B virus (HBV) in 1 patient (0.1%), while the incidence of herpes zoster infections was 3.6% (31). Possible explanations for the occurrence of potentially severe and/or recurrent viral infections include impairment of natural killer cells (NKs), which have a key role in controlling herpes infections, especially when T cells are low and a reduction of T regulatory cells (Tregs) protective against virus occurs.

Also, in the PV setting, two randomized phase 3 trials which compared ruxolitinib vs. best Supportive Care (RESPONSE and RESPONSE-2 studies) (32, 33) showed that the rates of herpes zoster infections during extended treatment were higher in patients receiving ruxolitinib as respect to best available therapy. In RESPONSE at 80 weeks, the rate per 100 patient-years of exposure was 5.4 vs. none (34). Most herpes zoster infections were grade 1 or 2 and were resolved without sequelae. Similarly, week 80 data in RESPONSE-2 (35) showed that the all-grade exposure-adjusted rate of herpes zoster infection per 100 patient-years of exposure was 3.8 in patients originally randomized to ruxolitinib, 7.5 in patients receiving ruxolitinib after crossover, and none in the best available therapy arm. No pneumonia or tuberculosis reactivation was observed in the ruxolitinib group. A summary of exposure-adjusted rates of immunosuppressive events during long-term treatment with ruxolitinib in the RESPONSE studies is presented in Table 2.

**Table 2:** Common immunosuppressive events during long-term treatment with ruxolitinib: Data from week 80 analyses of the RESPONSE and RESPONSE-2 trials in patients with polycythemia vera.

	**Ruxolitinib, randomized (*****n*** **=** **110)**		Ruxolitinib, crossover^*^ **(*****n*** **=** **98)**		**Best available therapy (*****n*** **=** 111)^**^	
	**All grades**	**Grade 3 or 4**	**All grades**	**Grade 3 or 4**	**All grades**	**Grade 3 or 4**
**RESPONSE (Verstovsek S et al. Haematologica 2016; 101:821-9)**						
All infections	29.4	4.0	27.8	5.4	58.4	4.1
Herpes zoster	5.3	0.9	5.4	0.7	0	0
Pyrexia	5.3	0	5.4	0.7	6.8	0
Nasopharyngitis	5.7	0	6.1	0	12.2	0
	**Ruxolitinib, randomized (*****n*** **=** **74)**		Ruxolitinib, crossover^*^ **(*****n*** **=** **58)**		**Best available therapy (*****n*** **=** **75)**	
	**All grades**	**Grade 3 or 4**	**All grades**	**Grade 3 or 4**	**All grades**	**Grade 3 or 4**
**RESPONSE-2 (Griesshammer M et al. Ann Hematol 2018; 97:1591–1600)**						
Infections and infestations	24.9	2.3	29.9	1.5	33.7	3.7
Upper respiratory tract infection	2.3	0	3.0	0	13.1	0
Nasopharyngitis	3.8	0	9.0	0	3.7	0
Influenza	4.5	0.8	1.5	0	9.4	1.9
Anemia	14.3	0	17.9	0	3.7	1.9
Thrombocytopenia	1.5	0	4.5	0	15.0	5.6
Pneumonia	0.8	0	0	0	1.9	1.9
Herpes zoster	3.8	0	7.5	0	0	0
Urinary tract infection	1.5	0	1.5	0	0	0
Urosepsis	0.8	0	0	0	0	0
Septic shock	0	0	0	0	1.9	1.9

*Data are exposure-adjusted rates per 100 patient years, regardless of relationship to study drug*.

* *Patients randomized to best available therapy were allowed to crossover to receive ruxolitinib after 32 (RESPONSE) or 28 (RESPONSE-2) weeks if they did not meet the primary end or for safety-related reasons*.

** *One patient was randomized to best available therapy but did not receive study treatment*.

*RESPONSE, Randomized Study of Efficacy and Safety in Polycythemia Vera With JAK Inhibitor INCB018424 vs. Best Supportive Care*.

Details of the characteristics of the randomized phase III trials of ruxolitinib in MF or PV can be found in Supplementary Table S1.

With the increasing use of ruxolitinib in clinical practice, there have also been reports of HBV reactivation and severe and uncommon infections including *Cryptococcus neoformans* pneumonia, toxoplasmosis retinitis, disseminated tuberculosis, and progressive multifocal leukoencephalopathy (36–42). Particularly, tuberculosis reactivation during ruxolitinib may be due to the impairment of dendritic cells (DCs) and IL-12 production, a key cytokine involved in the transcription of interferon (IFN)-gamma. In addition, ruxolitinib induces depression of T helper (Th)1 lymphocyte responses and production of IFN-gamma and tumor necrosis factor (TNF)-alpha (43).

**Figure 1:**
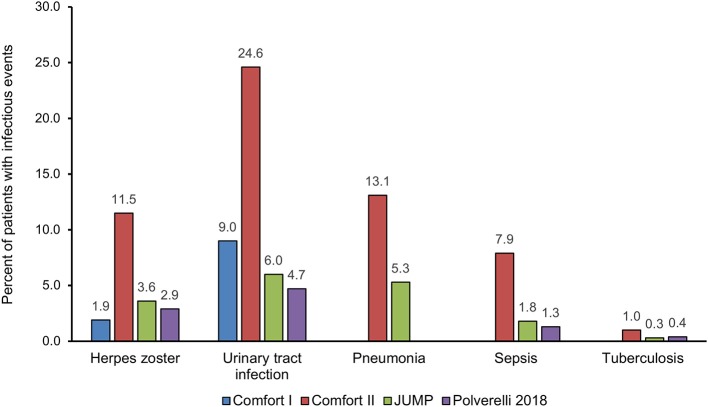
Distribution of the most important infectious events, regardless of relationship to study drug, in the principal studies of ruxolitinib in myelofibrosis.

A recent multicenter retrospective study on a large cohort of 446 patients with MF treated with ruxolitinib (44) showed that almost 30% of patients, with a median exposure to ruxolitinib of 23.5 months, experienced infectious events (incidence rate of 17 cases per 100 patients/year). Infections involving the respiratory tract were predominant and accounted for 50% of all infections reported. The rate of infections tended to decrease with time. Respiratory tract infections were more frequently observed (81 events, 50%), and bacteria were the most frequent etiological agents (68.9%). However, viral (14.9%) and fungal infections (2.5%) were also observed. Multivariate analysis found that a previous infectious event and a high-risk score according to the International Prognostic Score System (45) correlated significantly with a greater risk of infections. Of note, splenomegaly reduction by ≥50% from baseline to 3 months was significantly associated with longer infection-free survival. Figure 1 summarizes the incidence of the most important infectious events, regardless of relationship to study drug, in the principal studies of ruxolitinib in MF.

An increased risk of lymphoma during treatment with ruxolitinib or discontinuation of ruxolitinib has been suggested by sporadic reports (46, 47). Recently, four cases of aggressive lymphoma were reported during treatment among 69 MPNs patients receiving ruxolitinib (5.8% rate of lymphoma development) (22). Among MPNs patients from the same center who did not receive ruxolitinib (*n* = 557), two (0.36%) developed lymphoma. The analysis of an independent MPNs cohort (*n* = 929) found similar rates of lymphoma development (3.51% in patients receiving JAK1/2 inhibitor therapy and 0.23% in patients receiving conventional therapy). The lymphomas were found to have developed from pre-existing B-cell clones. In mice lacking STAT1 and treated with ruxolitinib, the phenotype of coexisting MPNs and B-cell transformation was reproduced, suggesting that the reduced ability of T lymphocytes and NKs to eliminate malignant hematopoietic cells, caused by STAT1 deficiency, could facilitate the development of a B-cell lymphoma (22). However, in a recent large database review, that included 2,583 patients with MPNs (comprised of 1,617 patients with MF) no statistically significant difference in the incidence of a subsequent lymphoma diagnosis in patients with MPNs when comparing those who received prior JAK inhibitor therapy vs. those who did not, was found (48).

Five-year efficacy data on ruxolitinib in MF showed 17.1% of patients on ruxolitinib went on to have basal cell carcinomas (BCCs) or squamous cell carcinomas (SCCs) compared with only 2.7% of patients on best available therapy (29). Skin cancers occurring in patients treated with ruxolitinib have been reported in the dermatology literature to display more aggressive biological behavior and metastatic potential (49). In a recent large international case-control study, including 1,881 patients with MPNs, a significantly higher risk of non-melanoma skin cancer (NMSC) was documented for ruxolitinib, suggesting that JAK1-JAK2 inhibitor may act as immunosuppressive agent (23).

In the PV setting, in the RESPONSE study, the rates of NMSC per 100 patient-years of exposure seemed to be higher in patients receiving ruxolitinib, as respect to best available therapy (4.4 vs. 2.7). NMSC cancers were observed in the originally randomized ruxolitinib arm, mainly in patients with a history of NMSC or precancer; however, exposure-adjusted rates at the time of this analysis were generally similar between the originally randomized ruxolitinib and best available therapy arms (34).

Finally, the potent anti-inflammatory and immunosuppressive properties of ruxolitinib can be exploited for the management of severe conditions including autoimmune or inflammatory diseases (50, 51) and corticosteroid-resistant GVHD (24–28). Preliminary studies with topical ruxolitinib demonstrated improvement in psoriasis compared to treatment with placebo or other topical approved therapies, but it was not a sustained improvement after discontinuation (52); importantly, systemic absorption was minimal, and there was no evidence of systemic toxicity. The potential efficacy of ruxolitinib is also being investigated in the treatment of alopecia areata and vitiligo, but these studies are still in their infancy (51, 53). A recent multicenter retrospective analysis of ruxolitinib as salvage treatment in patients with steroid-resistant acute or chronic GVHD found elevated overall response rates (>80%) and 6-month survival rates ranging from 79% (acute disease) to 97% (chronic disease), despite the fact that the patients had been heavily pretreated for GVHD, and their condition was generally severe (26). Based on the evidence supporting a role in preventing GVHD, ruxolitinib received in 2016 Breakthrough Therapy Designation from the US FDA for the treatment of GVHD (54).

## Cellular Targets of the Immunosuppressive Activity of Ruxolitinib

**Figure 2:**
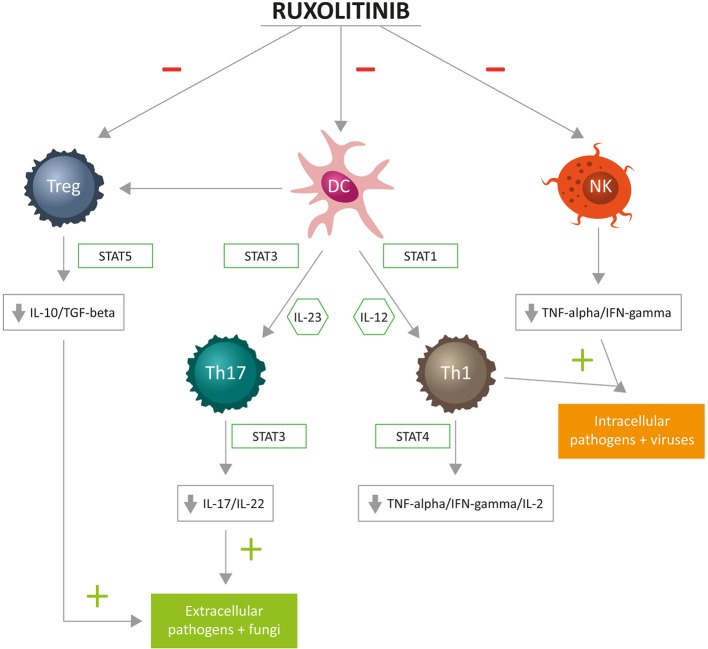
Cellular targets of immunosuppressive activity of ruxolitinib: T helper (Th)1 cells differentiate in the presence of interleukin (IL)-12 and are committed through STAT1. Fully committed Th1 cells produce interferon (IFN)-gamma through STAT4, of key importance for cell-mediated immune responses against intracellular bacteria and viruses. Th17 cells differentiate in the presence of IL-23 and are committed through STAT3. Fully committed Th17 produce IL-17 and IL-22 through STAT3, with a principal role for cell-mediated immune responses against extracellular bacteria and fungi. Tregs, through STAT5, produce IL-10 and transforming growth factor (TGF)-beta, contributing to immunosurveillance.

Available evidence suggests that ruxolitinib acts on cellular components of both innate and adaptive immunity (5). The innate immune system ensures front-line host defense and includes anatomic barriers, antimicrobial molecules, such as complement and cellular components: eosinophils, neutrophils, mast cells, basophils, NKs, monocytes/macrophages, and DCs; the adaptive immune system comprises CD4+ T-helper lymphocytes (Th1, Th2, Th17) and Tregs, CD8+ cytotoxic lymphocytes, and B lymphocytes (55). Figure 2 summarizes the complex relationship between ruxolitinib and the immune “orchestra.” In the following paragraphs, we will briefly discuss studies reporting the effects of ruxolitinib on NKs, DCs, and T cells. Potential additional immunological targets of ruxolitinib are also briefly discussed.

### Natural Killer Cells

NKs are the main effectors of the innate immune system. They are large lymphocyte-like cells with distinctive cytotoxic granules and lack antigen-specific receptors (55). They can detect and exert lytic activity on certain virus-infected cells, as well as on malignant cells, and are therefore important components also of cancer immunosurveillance mechanisms. NKs also produce cytokines, mainly IFN-gamma, and TNF-alpha, which modulate the differentiation of cells involved in adaptive immunity (Th1) and induce the maturation of DCs. The process leading to the development and differentiation of NKs from lymphoid precursors is mainly regulated by IL-15 and IL-2 with the involvement of the JAK1-JAK3/STAT5 pathway (5).

A recent study provided a detailed analysis of the influence of ruxolitinib on the biology of NKs by comparing the effects of JAK1/2 inhibition on this cell type in a cohort of 28 MPNs patients with or without ruxolitinib treatment and 24 healthy individuals (56). The analysis included cell frequency, receptor expression, proliferation, immune synapse formation, and cytokine signaling. The study found a reduction in NKs number in ruxolitinib-treated patients that was linked to the appearance of clinically relevant infections. An increase in the ratio of immature/mature NKs suggested that the observed reduction was due to impaired NKs maturation. *In vitro* experiments showed that the observed reduction in killing activity was caused by impairment in lytic synapse formation with target cells. The effect was reversible, as NKs function was restored upon ruxolitinib discontinuation. The authors suggested that ruxolitinib was likely inhibiting the JAK1-JAK3/STAT5 pathway downstream of the IL-2- and IL-15 receptors. In a study that compared the effects of ruxolitinib and the JAK2-specific inhibitor pacritinib (TG101348) on the function and activation of NKs, ruxolitinib was shown to completely block IL-2, IL-15, and the phosphorylation of STAT5 mediated by DCs, along with the capacity of NKs to secrete IFN-gamma or lyse target cells (57). In contrast, pacritinib treatment of stimulated NKs resulted in substantially less functional impairment. Therefore, selective JAK2 blockade ensures greater NKs activity than non-selective targeting of both JAK1 and JAK2.

Lastly, Ankathatti and Hu (58) showed, in a study with human cell lines, that small molecule blockers, including ruxolitinib, for JAK-STAT pathway, significantly inhibited cytokine secretion by macrophages activated by viral infectious trigger. Usually, macrophages respond to viral infection by releasing soluble mediators, helping in the recruitment of innate, and adaptative effector cells. In this process, they recruit NK cells to the site of infection and with DCs help, regulate NK maturation, and NK killing activity through production of type I interferons, Il-12, and IL-15. The authors demonstrated that NK cells activated by cytokines produced by activated macrophages could be inhibited in their killing activity by ruxolitinib, because of block of IL-12 and IL-15 production by macrophages.

### Dendritic Cells

DCs are important antigen-presenting cells (APC) and phagocytic cells; as macrophages, they are responsible for presenting antigens to T lymphocytes and initiating adaptive immunity (55). DCs are the unique propriety of inducing the differentiation of naïve T cells in helper and effector T cells. More in detail, the induction of an adaptive immune response initiates following the ingestion of a pathogen by an immature DCs in the infected tissue. Upon ingestion of a pathogen, DCs become activated and migrate to a nearby lymph node where they prime T lymphocytes toward effector or regulatory responses. Activated DCs secrete cytokines that influence both innate and adaptive immune responses and play, therefore, a key role in determining whether, and how, the immune system responds to an infectious agent. In particular, DCs control the adaptative response that leads to the production of IL-12 and IL-23, cytokines that drive Th1 and Th17 lymphocytes phenotypes. More specifically, Th1 cells produce cytokine IFN-gamma, IL-2, and TNF-alpha. TNF-alpha improves Th1 against intracellular pathogens. Th17 secrete IL-17 and IL-22, key cytokines against extracellular bacteria. More mature DCs mediate the induction of T-regs.

The important study by Heine and coworkers on the effects of ruxolitinib on DCs biology demonstrated *in vitro* and *in vivo* that JAK1/2 inhibitor therapy affects cell differentiation, tissue migration and function of DCs resulting in impaired induction of allogenic or antigen-specific T-cell responses, including viral clearance. These findings were among the first to clearly show that ruxolitinib has potent anti-inflammatory and immunomodulating activity, and to provide a possible explanation for the increased infection rates reported in MPNs patients exposed to ruxolitinib (59).

A more recent study analyzed the impact of ruxolitinib on the migration of DCs with a focus on short-term effects and the identification of potential target molecules mediating these effects (60). The study identified Rho-associated coiled-coil kinase 1 (ROCK) as a target of ruxolitinib. Notably, this protein regulates the reorganization and contraction of cellular actin-myosin filaments and plays an important role in the migration of DCs. Via interference with ROCK activation, ruxolitinib profoundly impairs DCs migration; subsequently, the loss of trafficking DCs may lead to reduced activation of T cells in draining lymph nodes and might, therefore, explain the abundance of these proinflammatory cells from the blood of MPNs patients after ruxolitinib treatment (61).

### CD4+ T Cells

CD4+ T cells, which differentiate from naïve T cells, are a heterogeneous cell population with a central role in adaptive immunity. They mainly act via the secretion of cytokines and chemokines that induce and/or recruit target cells and are able to adopt a series of distinct differentiated states including T-helper and Treg cell types (61, 62). Tregs suppress autoreactive lymphocytes, prevent autoimmune disease, and control innate and adaptive immune responses (62). Tregs also control viral, fungal, and protozoan infections. They produce inhibitory cytokines such as IL-10 and TGF-beta that promote fibrosis, affect the function and induce apoptosis of T-effector lymphocytes. Given the importance of JAK-STAT signaling in regulating the fate and function of CD4+ T cells, a study attempted to define the effect of ruxolitinib on the function of this cell subset in MPNs patients (61). The study, which involved both *in vitro* and *in vivo* experiments, highlighted a reduction in total CD3+ cells after 3 weeks of ruxolitinib treatment in MPNs patients. The number of Th1, Th17, and Tregs were also reduced, a result that was validated also *in vitro*. In line with these findings, inflammatory cytokines, including TNF-alpha, IL-5, IL-6, and IL-1B, were found to be significantly downregulated in T cells isolated from the patients. Notably, ruxolitinib did not interfere with the T cell receptor signaling pathway, but inhibited IL2-dependent STAT5 activation. The reduction in the number of Tregs induced by ruxolitinib was in line with the results of a previous study conducted by Massa et al. in 18 patients with MF to define the impact of JAK1/2 inhibition on immunosurveillance (63). Massa et al. showed that the treatment with ruxolitinib resulted in a long-lasting reduction in circulating Tregs. Dose reduction or withdrawal of the drug failed to restore the Treg subset to values comparable to those of the control, suggesting that the effect of ruxolitinib was irreversible. The authors suggested that the severe reduction in circulating Tregs observed in their patients exposed to ruxolitinib may constitute a major cause of immunosurveillance disruption.

A recent study investigated the frequency and function of CD4+ T cell subsets in 50 MPN patients at baseline as well as during treatment with either ruxolitinib or fedratinib (SAR302503; TG101348), a selective JAK2 inhibitor (64). The study showed, for the first time, that Tregs are reduced in MPNs patients compared to healthy controls and that this decrease becomes more pronounced following JAK inhibitor therapy. However, after 6 months, responders to treatment with JAK inhibitor therapy displayed an increased number of Th17, which are known to secrete IL-17 and to play a key role in autoimmune disease, as well as in tumor immunosurveillance. This result could suggest a potential polarization from a Treg /Th1 to a Th17 phenotype. Functional “silencing” of Th cells, both *in vivo* and *in vitro*, and the blockade of proinflammatory cytokines from these cells were also observed. Thus, treatment with JAK inhibitor therapy had both short- and long-term effects on CD4+ T cells in MPNs. The short-term effect involved a reduction in Treg number, Th silencing, and reduction of cytokine secretion. The long-term effect, on the other hand, resulted in immune response polarization toward a Th17-type response. Thanks to this repolarization effect, according to the authors, immunosurveillance against the malignant clone could be resumed.

### Additional Immunological Targets of Ruxolitinib

Given the complexity of the signaling pathways modulated by JAK1 and JAK2, and the wide range of immune system components affected, it can reasonably be expected that JAK1/2 inhibitor therapy with ruxolitinib will have other cellular targets beside those described in the previous sections. An important open question concerns the effect of ruxolitinib on the B cell population and, in particular, on antibody production. Optimal B cell activity requires a complex interplay of inputs from a variety of T cells, including T effector cells and Tregs (5). Establishing the impact of JAK inhibition on antibody production is currently difficult to predict yet crucial when considering the need for optimal defense against pathogenic infections. No evidence of antibody deficiency has emerged from the COMFORT-I and II studies (15, 18, 29). With regard to possible abnormalities in B cells associated with MPNs, available evidence shows that the mutant clone may also include lymphoid-derived cells (5). A number of reports in small groups of patients have shown that a few patients with chronic MPNs carry the *JAK2* V617F mutation in both B- and T-lymphocytes, suggesting that the mutation occurred early, in lympho-myeloid progenitor cells (65, 66).

Other potential targets of ruxolitinib include neutrophils and macrophages, two important components of innate immunity. Neutrophil activation is mediated by JAK1/2 signaling (26). Furthermore, it has been shown that neutrophils have a key role in the pathogenesis of acute GVHD (26, 67, 68). As for macrophages, ruxolitinib was shown to prevent the up-regulation of various proinflammatory cytokines in human macrophages (69). Ruxolitinib impairs cytokine expression by inhibiting LPS/TLR4/IFN beta signaling pathway. Cytokine repression contributes to the anti-inflammatory effect of ruxolitinib. However, earlier data suggested that the inhibition of JAKs may increase the inflammatory potential of macrophages exposed to TLR4 agonists, which potently stimulate cytokine production (70).

## Conclusions

The substantial clinical benefits and efficacy associated with the use of JAK1/2 inhibitor therapy need to be balanced against the multiple effects on components of both innate and adaptive immunity, including NKs, DCs, and T cells (Th and Tregs). Ruxolitinib impairs several cytokines, modulates DCs function and T cell response and reduces NKs levels in MPNs patients, which may lead to increased risk of opportunistic infection and reactivation of latent infections. Age and comorbidities, treatments (such as steroids) and environmental exposure may influence the risk of infections, that may occur early and late after treatment.

Thus, prior to initiation of therapy with ruxolitinib and other JAK inhibitors, patient counseling, assessment of risk factors for tuberculosis reactivation, screening for previous hepatitis B and C exposure, anti-infective prophylaxis, along with particular caution in patients who are already immunocompromised, are highly recommended (12, 13, 43).

Surveillance of HBV markers and viral load are important due to the high incidence of latent HBV and reactivation during steroid or immunosuppressive drugs. Prompt antiviral prophylaxis should be considered in hematological patients with a high risk of HBV reactivation according to treatment guidelines (71). Similarly, HCV carriers must be identified and treated according to viral load.

Also, active surveillance of second solid neoplasia and hematological diseases, including high-grade lymphomas, should always be performed during therapy. Accordingly, current ruxolitinib prescriber information labels warn of the risk of NMSC, and performing periodic skin examinations is recommended patients (23). Also, the detection of a preexisting B-cell clone may identify individuals at risk for lymphoma development (22).

More robust data are necessary to answer the question of possible immune derangement of ruxolitinib treatment in MPNs patients. Although its immunosuppressive properties should not be forgotten, ruxolitinib remains a cornerstone in the treatment of BCR-ABL negative MPNs, where its anti-inflammatory activity is a benefit for the patient and especially in MF where symptom control, reduction of splenomegaly and possibly improvement of survival may be obtained. Finally, the potent anti-inflammatory and immunosuppressive activity of ruxolitinib is proving valuable for the management of severe and life-threatening conditions like GVHD in the allogeneic stem cell transplantation setting.

## Author Contributions

EE and GP conceived the manuscript and prepared the first draft. All authors critically revised the article for important intellectual content. All authors gave final approval of the version to be published, and agree to be accountable for all aspects of the work in ensuring that questions related to the accuracy or integrity of any part of the work are appropriately investigated and resolved.

### Conflict of Interest

EE has participated at advisory boards for Novartis. CB has participated at advisory boards for Abbvie, BMS, Celgene, Incyte, Janssen, Novartis. FP has received honoraria from Novartis. GP has received honoraria from Amgen, Celgene, Novartis and has participated at advisory boards for Celgene, Janssen, Novartis. The remaining author declares that the research was conducted in the absence of any commercial or financial relationships that could be construed as a potential conflict of interest.

## Abbreviations

APC: Antigen-presenting cells
BCCs: Basal cell carcinomas
CALR: Calreticulin
COMFORT: Controlled Myelofibrosis Study with Oral JAK Inhibitor Treatment
DCs: Dendritic cells
EMA: European Medicines Agency
ET: Essential thrombocythemia
FDA: U.S. Food and Drug Administration
GVHD: Graft versus host disease
HBV: Hepatitis B virus
HCV: Hepatitis C virus
IC50: Half maximal inhibitory concentration
IFN: Interferon
IL: Interleukin
JAK: Janus kinase
JUMP: JAK Inhibitor Ruxolitinib in Myelofibrosis Patients
MF: Myelofibrosis
MPL: Thrombopoietin receptor gene
MPNs: Myeloproliferative neoplasms
NKs: Natural killer cells
NMSC: Nonmelanoma skin cancer
PPV/PET-MF, Post-polycythemia vera: and post-essential thrombocythemia MF
PV: Polycythemia vera
RESPONSE: Randomized Study of Efficacy and Safety in Polycythemia Vera With JAK Inhibitor INCB018424 Versus Best Supportive Care
ROCK: Rho-associated coiled-coil kinase
SCC: Squamous cell carcinoma
STAT: Signal transducer and activator of transcription
Th: T helper
TNF: Tumor necrosis factor
Treg: T regulatory cells.
