# Supplementary material for: Mechanisms Underlying the Anti-inflammatory and Immunosuppressive Activity of Ruxolitinib
Source: Front Oncol. 2019 Nov 7;9:1186. doi: 10.3389/fonc.2019.01186 (PMC6854013; doi:10.3389/fonc.2019.01186)
Supplement: Supplementary file 1 [file Data_Sheet_1.PDF]

Supplementary Table S1 Characteristics of phase III randomized controlled trials of ruxolitinib in patients with myelofibrosis or polycythemia vera.

Study	Study population	Total number of patients	Intervention (N)	Comparison (N)	Treatment duration (randomization phase)	Follow-up period (extended phase)
COMFORT-I (Verstovsek et al., 2017)	Myelofibrosis (primary and post-PV or ET)	309	Ruxolitinib 15 mg or 20 mg bid po based on Plt level (n = 155)	Placebo (n = 154)	24 weeks	5 years
COMFORT-II (Harrison et al., 2016)	Myelofibrosis (primary and post-PV or ET)	219	Ruxolitinib 15 mg or 20 mg bid po based on Plt level (n = 146)	Best available therapy (n = 73)	48 weeks	5 years
RESPONSE (Verstovsek et al., 2016)	Polycythemia vera	222	Ruxolitinib 10 mg (starting dose) bid po (n = 110)	Best available therapy (n = 112)	32 weeks	80 weeks
RESPONSE-2 (Griesshammer et al., 2018)	Polycythemia vera	149	Ruxolitinib 10 mg (starting dose) bid po (n = 74)	Best available therapy (n = 75)	28 weeks	80 weeks

PV, Polycythemia vera; *ET*, Essential thrombocythemia; *bid*, twice daily; *po*, orally; *Plt*, platelets;
COMFORT, Controlled Myelofibrosis Study with Oral JAK Inhibitor Treatment;
RESPONSE, Randomized Study of Efficacy and Safety in Polycythemia Vera With JAK Inhibitor INCB018424 Versus Best Supportive Care.

References:

Griesshammer, M., Saydam, G., Palandri, F., Benevolo, G., Egyed, M., Callum, J., et al. (2018). Ruxolitinib for the treatment of inadequately controlled polycythemia vera without splenomegaly: 80-week follow-up from the RESPONSE-2 trial. Ann Hematol 97(9), 1591-1600. doi: 10.1007/s00277-018-3365-y.

Harrison, C.N., Vannucchi, A.M., Kiladjian, J.J., Al-Ali, H.K., Gisslinger, H., Knoops, L., et al. (2016). Long-term findings from COMFORT-II, a phase 3 study of ruxolitinib vs best available therapy for myelofibrosis. Leukemia 30(8), 1701-1707. doi: 10.1038/leu.2016.148.

Verstovsek, S., Mesa, R.A., Gotlib, J., Gupta, V., DiPersio, J.F., Catalano, J.V., et al. (2017). Long-term treatment with ruxolitinib for patients with myelofibrosis: 5-year update from the randomized, double-blind, placebo-controlled, phase 3 COMFORT-I trial. J Hematol Oncol 10(1), 55. doi: 10.1186/s13045-017-0417-z.

Verstovsek, S., Vannucchi, A.M., Griesshammer, M., Masszi, T., Durrant, S., Passamonti, F., et al. (2016). Ruxolitinib versus best available therapy in patients with polycythemia vera: 80-week follow-up from the RESPONSE trial. Haematologica 101(7), 821-829. doi: 10.3324/haematol.2016.143644.
